# Bis{1-[4-(benz­yloxy)phen­yl]-4,4,4-tri­fluoro­butane-1,3-dionato(1−)}dipyri­dine­cobalt(II)

**DOI:** 10.1107/S1600536811043157

**Published:** 2011-10-29

**Authors:** Ling Fan, Yongzhou Chen, Xianhong Wei, Guodong Yin

**Affiliations:** a College of Chemistry and Environmental Engineering, Hubei Normal University, Huangshi 435002, People’s Republic of China

## Abstract

In the title compound, [Co(C_17_H_12_F_3_O_3_)_2_(C_5_H_5_N)_2_], the Co^II^ ion is situated on a twofold rotation axis, coordinated by four O atoms from two 1-[4-(benz­yloxy)phen­yl]-4,4,4-trifluoro­butane-1,3-dionate(1−) (*L*) ligands and two N atoms from two pyridine ligands in a distorted octa­hedral geometry. The two pyridine rings form a dihedral angle of 84.63 (7)°. The two benzene rings in *L* are twisted at 58.83 (5)°. Weak inter­molecular C—H⋯F hydrogen bonds consolidate the crystal packing.

## Related literature

For the crystal structures of other complexes of transition metal ions with β-diketonate ligands, see: Melnik *et al.* (1999 ▶); Soldatov *et al.* (2003 ▶); Youngme *et al.* (2007 ▶); Feng (2002 ▶).
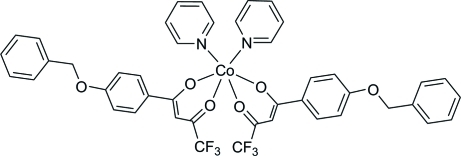


## Experimental

### 

#### Crystal data

[Co(C_17_H_12_F_3_O_3_)_2_(C_5_H_5_N)_2_]
*M* *_r_* = 859.66
Monoclinic, 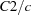

*a* = 16.5435 (11) Å
*b* = 10.7359 (7) Å
*c* = 23.0706 (14) Å
β = 107.333 (1)°
*V* = 3911.5 (4) Å^3^
*Z* = 4
Mo *K*α radiation
μ = 0.52 mm^−1^
*T* = 293 K
0.20 × 0.20 × 0.06 mm

#### Data collection

Bruker SMART APEX CCD area-detector diffractometer
Absorption correction: multi-scan (*SADABS*; Sheldrick, 1996 ▶) *T*_min_ = 0.903, *T*_max_ = 0.970
21577 measured reflections
4453 independent reflections
2849 reflections with *I* > 2σ(*I*)
*R*_int_ = 0.051

#### Refinement

*R*[*F* ^2^ > 2σ(*F* ^2^)] = 0.051
*wR*(*F* ^2^) = 0.151
*S* = 1.09
4453 reflections
267 parameters
H-atom parameters constrained
Δρ_max_ = 0.46 e Å^−3^
Δρ_min_ = −0.40 e Å^−3^

### 

Data collection: *SMART* (Bruker, 2001 ▶); cell refinement: *SAINT* (Bruker, 1999 ▶); data reduction: *SAINT*; program(s) used to solve structure: *SHELXS97* (Sheldrick, 2008 ▶); program(s) used to refine structure: *SHELXL97* (Sheldrick, 2008 ▶); molecular graphics: *SHELXTL* (Sheldrick, 2008 ▶); software used to prepare material for publication: *SHELXTL*.

## Supplementary Material

Crystal structure: contains datablock(s) I, global. DOI: 10.1107/S1600536811043157/cv5178sup1.cif

Structure factors: contains datablock(s) I. DOI: 10.1107/S1600536811043157/cv5178Isup2.hkl

Additional supplementary materials:  crystallographic information; 3D view; checkCIF report

## Figures and Tables

**Table 1:** Hydrogen-bond geometry (Å, °)

*D*—H⋯*A*	*D*—H	H⋯*A*	*D*⋯*A*	*D*—H⋯*A*
C2—H2⋯F2^i^	0.93	2.62	3.385 (3)	140
C19—H19⋯F3^ii^	0.93	2.63	3.348 (3)	134
C7—H7*B*⋯F1^ii^	0.97	2.62	3.507 (3)	152

Symmetry codes: (i) 
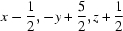
; (ii) 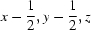.

**Fig. 1.**
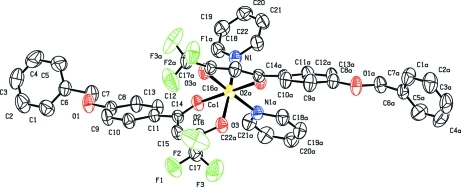
The title complex with the atom-numbering scheme [symmetry code: (a) -x + 1, y, -z + 3/2]. Displacement ellipsoids are drawn at the 30% probability level.

## supplementary crystallographic information

### Comment

Coordination complexes of metal ions with β-diketonate ligands have proven useful in a wide range of application (Melnik *et al.*, 1999; Soldatov *et al.*, 2003; Youngme *et al.*, 2007). The modification of the steric properties of the β-diketonate ligand is significant as a means of controlling the ability for additional ligand binding. Herein, we report the crystal structure of the title compound (I), which is a pyridine adduct of cobalt(II) complex with ligand 1-(4-(benzyloxy)phenyl)-4,4,4-trifluorobutane-1,3-dione (Fig. 1).

In (I), the cobalt(II) is six-coordinated by four O atoms of the ligands and two N atoms of pyridines, giving an octahedral geometry. Atoms O2, O3, O2a and N1 form an equatorial plane, while N1a and O3a occupy the axial positions. The coordinating bond lengths [Co1—N1 = 2.158 (2) Å, Co1—O2 = 2.055 (2) Å, Co1—O3 = 2.088 (2) Å] are in good agree with those found in other Co^II^β-diketonate complex (Feng, 2002). The bond lengths of C14—C15 and C15—C16 are 1.405 (3) and 1.388 (3) Å respectively, which indicate the carbon-carbon double bond in the enol form of ligand is close to the trifluoromethyl group. The angles of O2—Co1—O2a and O3—Co1—N1 are 176.53 (10)° and 176.47 (7)° respectively, suggesting almost coplanar nature of O2, O3, O2a, N1 and Co1 (largest deviation 0.06 Å). Two pyridyl rings are nearly perpendicular with the dihedral angle of 84.63 (7)°. The dihedral angle between two phenyl rings of C1–C6 and C8–C13 in each independent ligand is 58.83 (2)°. Weak intermolecular C—H···F hydrogen bonds (Table 1) consolidate the crystal packing.

### Experimental

To a hot ethanol solution (25 ml) of the ligand (644 mg, 2.0 mmol) and pyridine (316 mg, 4.0 mmol) was added slowly to 20 ml water solution of cobalt(II) acetate tetrahydrate (250 mg, 1.0 mmol). The mixture was stirred for another 6 h. After filtration, the red solution was allowed to stand at room temperature. Red block-shaped crystals suitable for X-ray analysis were obtained after several days.

### Refinement

C-bound H atoms were positioned geometrically (C—H 0.93-0.97 Å), and refined as riding, with *U*_iso_(H) = 1.2*U*_eq_(C).

### Crystal data

**Table:**

[Co(C_17_H_12_F_3_O_3_)_2_(C_5_H_5_N)_2_]	*F*(000) = 1764
*M**_r_* = 859.66	*D*_x_ = 1.460 Mg m^−^^3^
Monoclinic, *C*2/*c*	Mo *K*α radiation, λ = 0.71073 Å
Hall symbol: -C2yc	Cell parameters from 3520 reflections
*a* = 16.5435 (11) Å	θ = 2.3–21.5°
*b* = 10.7359 (7) Å	µ = 0.52 mm^−^^1^
*c* = 23.0706 (14) Å	*T* = 293 K
β = 107.333 (1)°	Plate, red
*V* = 3911.5 (4) Å^3^	0.20 × 0.20 × 0.06 mm
*Z* = 4	

### Data collection

**Table:**

Bruker SMART APEX CCD area-detector diffractometer	4453 independent reflections
Radiation source: fine-focus sealed tube	2849 reflections with *I* > 2σ(*I*)
graphite	*R*_int_ = 0.051
φ and ω scans	θ_max_ = 27.5°, θ_min_ = 2.3°
Absorption correction: multi-scan (*SADABS*; Sheldrick, 1996)	*h* = −21→21
*T*_min_ = 0.903, *T*_max_ = 0.970	*k* = −13→13
21577 measured reflections	*l* = −29→28

### Refinement

**Table:**

Refinement on *F*^2^	Primary atom site location: structure-invariant direct methods
Least-squares matrix: full	Secondary atom site location: difference Fourier map
*R*[*F*^2^ > 2σ(*F*^2^)] = 0.051	Hydrogen site location: inferred from neighbouring sites
*wR*(*F*^2^) = 0.151	H-atom parameters constrained
*S* = 1.09	*w* = 1/[σ^2^(*F*_o_^2^) + (0.0783*P*)^2^] where *P* = (*F*_o_^2^ + 2*F*_c_^2^)/3
4453 reflections	(Δ/σ)_max_ < 0.001
267 parameters	Δρ_max_ = 0.46 e Å^−^^3^
0 restraints	Δρ_min_ = −0.40 e Å^−^^3^

### Special details

**Table:**

Geometry. All e.s.d.'s (except the e.s.d. in the dihedral angle between two l.s. planes) are estimated using the full covariance matrix. The cell e.s.d.'s are taken into account individually in the estimation of e.s.d.'s in distances, angles and torsion angles; correlations between e.s.d.'s in cell parameters are only used when they are defined by crystal symmetry. An approximate (isotropic) treatment of cell e.s.d.'s is used for estimating e.s.d.'s involving l.s. planes.
Refinement. Refinement of *F*^2^ against ALL reflections. The weighted *R*-factor *wR* and goodness of fit *S* are based on *F*^2^, conventional *R*-factors *R* are based on *F*, with *F* set to zero for negative *F*^2^. The threshold expression of *F*^2^ > σ(*F*^2^) is used only for calculating *R*-factors(gt) *etc*. and is not relevant to the choice of reflections for refinement. *R*-factors based on *F*^2^ are statistically about twice as large as those based on *F*, and *R*-factors based on ALL data will be even larger.

### Fractional atomic coordinates and isotropic or equivalent isotropic displacement parameters (Å^2^)

**Table:**

	*x*	*y*	*z*	*U*_iso_*/*U*_eq_	
Co1	0.5000	0.98662 (4)	0.7500	0.04247 (19)	
C1	0.2027 (2)	1.0025 (3)	1.12451 (16)	0.0696 (9)	
H1	0.2575	1.0324	1.1408	0.083*	
C2	0.1457 (3)	1.0136 (3)	1.15707 (18)	0.0827 (11)	
H2	0.1624	1.0518	1.1950	0.099*	
C3	0.0658 (2)	0.9698 (3)	1.1347 (2)	0.0795 (11)	
H3	0.0276	0.9773	1.1570	0.095*	
C4	0.0423 (2)	0.9151 (4)	1.0796 (2)	0.0914 (12)	
H4	−0.0126	0.8849	1.0639	0.110*	
C5	0.0993 (2)	0.9034 (3)	1.04600 (15)	0.0755 (10)	
H5	0.0824	0.8650	1.0082	0.091*	
C6	0.17965 (17)	0.9480 (3)	1.06829 (13)	0.0492 (6)	
C7	0.24232 (19)	0.9304 (3)	1.03333 (14)	0.0583 (7)	
H7A	0.2892	0.8795	1.0566	0.070*	
H7B	0.2154	0.8880	0.9953	0.070*	
C8	0.32766 (16)	1.0515 (3)	0.98741 (12)	0.0477 (6)	
C9	0.35554 (17)	1.1677 (3)	0.97581 (13)	0.0556 (7)	
H9	0.3397	1.2382	0.9932	0.067*	
C10	0.40662 (16)	1.1793 (3)	0.93861 (12)	0.0506 (7)	
H10	0.4253	1.2579	0.9314	0.061*	
C11	0.43073 (14)	1.0755 (2)	0.91162 (10)	0.0404 (6)	
C12	0.40418 (16)	0.9594 (3)	0.92536 (12)	0.0485 (6)	
H12	0.4208	0.8885	0.9087	0.058*	
C13	0.35399 (17)	0.9467 (3)	0.96295 (12)	0.0505 (7)	
H13	0.3377	0.8678	0.9720	0.061*	
C14	0.47827 (15)	1.0839 (2)	0.86654 (11)	0.0409 (6)	
C15	0.52962 (16)	1.1877 (3)	0.86562 (12)	0.0500 (7)	
H15	0.5310	1.2517	0.8930	0.060*	
C16	0.57835 (15)	1.2005 (2)	0.82633 (11)	0.0447 (6)	
C17	0.63094 (19)	1.3182 (3)	0.83179 (14)	0.0593 (8)	
C18	0.35055 (18)	0.8074 (3)	0.74601 (13)	0.0572 (7)	
H18	0.3512	0.8481	0.7817	0.069*	
C19	0.29215 (18)	0.7144 (3)	0.72501 (14)	0.0635 (8)	
H19	0.2546	0.6929	0.7464	0.076*	
C20	0.28972 (18)	0.6538 (3)	0.67243 (14)	0.0606 (8)	
H20	0.2504	0.5911	0.6571	0.073*	
C21	0.34664 (19)	0.6878 (3)	0.64308 (15)	0.0643 (8)	
H21	0.3471	0.6476	0.6075	0.077*	
C22	0.40313 (18)	0.7815 (3)	0.66633 (14)	0.0594 (8)	
H22	0.4410	0.8039	0.6454	0.071*	
F1	0.63339 (13)	1.38935 (18)	0.87896 (9)	0.0861 (6)	
F2	0.60161 (18)	1.3903 (2)	0.78411 (10)	0.1257 (10)	
F3	0.71007 (13)	1.2954 (2)	0.83588 (14)	0.1230 (10)	
N1	0.40663 (13)	0.8423 (2)	0.71752 (9)	0.0465 (5)	
O1	0.27283 (12)	1.04931 (18)	1.02143 (9)	0.0609 (5)	
O2	0.47050 (12)	0.99242 (15)	0.83042 (9)	0.0498 (5)	
O3	0.58708 (11)	1.12821 (17)	0.78575 (8)	0.0514 (5)	

### Atomic displacement parameters (Å^2^)

**Table:**

	*U*^11^	*U*^22^	*U*^33^	*U*^12^	*U*^13^	*U*^23^
Co1	0.0523 (3)	0.0441 (3)	0.0385 (3)	0.000	0.0251 (2)	0.000
C1	0.070 (2)	0.086 (3)	0.062 (2)	−0.0213 (16)	0.0346 (17)	−0.0153 (17)
C2	0.102 (3)	0.093 (3)	0.071 (2)	−0.013 (2)	0.054 (2)	−0.0122 (19)
C3	0.083 (2)	0.086 (3)	0.094 (3)	0.0053 (19)	0.064 (2)	0.012 (2)
C4	0.0536 (19)	0.123 (3)	0.105 (3)	−0.015 (2)	0.0365 (19)	−0.001 (3)
C5	0.066 (2)	0.099 (3)	0.064 (2)	−0.0083 (18)	0.0222 (16)	−0.0125 (19)
C6	0.0547 (15)	0.0519 (16)	0.0497 (16)	0.0011 (13)	0.0290 (13)	0.0063 (13)
C7	0.0691 (18)	0.0594 (19)	0.0571 (18)	0.0001 (15)	0.0353 (15)	0.0042 (15)
C8	0.0497 (14)	0.0545 (17)	0.0469 (15)	−0.0009 (12)	0.0265 (12)	−0.0009 (13)
C9	0.0690 (17)	0.0505 (17)	0.0608 (18)	−0.0067 (14)	0.0400 (14)	−0.0142 (14)
C10	0.0596 (15)	0.0477 (16)	0.0550 (17)	−0.0094 (12)	0.0331 (13)	−0.0067 (13)
C11	0.0429 (13)	0.0462 (15)	0.0358 (13)	0.0003 (11)	0.0175 (11)	−0.0012 (11)
C12	0.0582 (15)	0.0455 (16)	0.0507 (16)	0.0075 (12)	0.0300 (13)	0.0005 (12)
C13	0.0642 (17)	0.0445 (16)	0.0553 (17)	0.0008 (13)	0.0369 (14)	0.0050 (13)
C14	0.0461 (13)	0.0462 (16)	0.0338 (13)	0.0015 (11)	0.0173 (11)	−0.0009 (11)
C15	0.0575 (15)	0.0524 (17)	0.0477 (15)	−0.0071 (13)	0.0275 (13)	−0.0100 (13)
C16	0.0461 (13)	0.0498 (16)	0.0423 (15)	−0.0029 (11)	0.0194 (11)	−0.0005 (12)
C17	0.0678 (18)	0.065 (2)	0.0558 (19)	−0.0145 (15)	0.0350 (15)	−0.0097 (16)
C18	0.0678 (18)	0.0599 (19)	0.0512 (16)	−0.0076 (14)	0.0288 (14)	−0.0048 (14)
C19	0.0602 (17)	0.069 (2)	0.070 (2)	−0.0099 (15)	0.0314 (15)	0.0027 (17)
C20	0.0567 (17)	0.0530 (18)	0.070 (2)	−0.0054 (13)	0.0151 (15)	−0.0021 (15)
C21	0.0685 (18)	0.063 (2)	0.0646 (19)	−0.0064 (15)	0.0252 (16)	−0.0197 (16)
C22	0.0652 (17)	0.0582 (19)	0.0625 (19)	−0.0050 (14)	0.0310 (15)	−0.0139 (15)
F1	0.1119 (15)	0.0785 (13)	0.0865 (13)	−0.0420 (11)	0.0579 (11)	−0.0326 (11)
F2	0.188 (3)	0.0923 (17)	0.0835 (15)	−0.0641 (16)	0.0195 (16)	0.0237 (13)
F3	0.0777 (13)	0.1051 (18)	0.212 (3)	−0.0376 (12)	0.0821 (16)	−0.0510 (18)
N1	0.0490 (11)	0.0471 (13)	0.0478 (13)	0.0008 (10)	0.0214 (10)	0.0004 (10)
O1	0.0777 (13)	0.0566 (12)	0.0695 (14)	−0.0051 (10)	0.0541 (11)	−0.0034 (10)
O2	0.0649 (11)	0.0468 (11)	0.0468 (11)	−0.0052 (8)	0.0305 (9)	−0.0048 (8)
O3	0.0592 (11)	0.0514 (11)	0.0547 (11)	−0.0069 (8)	0.0339 (9)	−0.0066 (9)

### Geometric parameters (Å, °)

**Table:**

Co1—O2	2.0553 (18)	C10—H10	0.9300
Co1—O2^i^	2.0553 (18)	C11—C12	1.389 (4)
Co1—O3	2.0877 (18)	C11—C14	1.482 (3)
Co1—O3^i^	2.0877 (18)	C12—C13	1.375 (3)
Co1—N1^i^	2.158 (2)	C12—H12	0.9300
Co1—N1	2.158 (2)	C13—H13	0.9300
C1—C6	1.369 (4)	C14—O2	1.269 (3)
C1—C2	1.374 (4)	C14—C15	1.405 (3)
C1—H1	0.9300	C15—C16	1.388 (3)
C2—C3	1.353 (5)	C15—H15	0.9300
C2—H2	0.9300	C16—O3	1.257 (3)
C3—C4	1.347 (5)	C16—C17	1.518 (4)
C3—H3	0.9300	C17—F3	1.307 (3)
C4—C5	1.395 (4)	C17—F2	1.314 (4)
C4—H4	0.9300	C17—F1	1.320 (3)
C5—C6	1.361 (4)	C18—N1	1.341 (3)
C5—H5	0.9300	C18—C19	1.373 (4)
C6—C7	1.503 (4)	C18—H18	0.9300
C7—O1	1.429 (3)	C19—C20	1.367 (4)
C7—H7A	0.9700	C19—H19	0.9300
C7—H7B	0.9700	C20—C21	1.363 (4)
C8—O1	1.365 (3)	C20—H20	0.9300
C8—C9	1.384 (4)	C21—C22	1.369 (4)
C8—C13	1.386 (4)	C21—H21	0.9300
C9—C10	1.378 (3)	C22—N1	1.336 (3)
C9—H9	0.9300	C22—H22	0.9300
C10—C11	1.392 (3)		
			
O2—Co1—O2^i^	176.53 (10)	C11—C10—H10	119.4
O2—Co1—O3	86.72 (7)	C12—C11—C10	117.7 (2)
O2^i^—Co1—O3	90.75 (7)	C12—C11—C14	119.0 (2)
O2—Co1—O3^i^	90.75 (7)	C10—C11—C14	123.2 (2)
O2^i^—Co1—O3^i^	86.72 (7)	C13—C12—C11	121.6 (2)
O3—Co1—O3^i^	86.54 (10)	C13—C12—H12	119.2
O2—Co1—N1^i^	92.64 (8)	C11—C12—H12	119.2
O2^i^—Co1—N1^i^	89.85 (7)	C12—C13—C8	119.9 (2)
O3—Co1—N1^i^	92.71 (8)	C12—C13—H13	120.1
O3^i^—Co1—N1^i^	176.47 (7)	C8—C13—H13	120.1
O2—Co1—N1	89.85 (7)	O2—C14—C15	123.2 (2)
O2^i^—Co1—N1	92.65 (8)	O2—C14—C11	116.3 (2)
O3—Co1—N1	176.47 (7)	C15—C14—C11	120.6 (2)
O3^i^—Co1—N1	92.71 (8)	C16—C15—C14	124.1 (2)
N1^i^—Co1—N1	88.26 (11)	C16—C15—H15	118.0
C6—C1—C2	120.8 (3)	C14—C15—H15	118.0
C6—C1—H1	119.6	O3—C16—C15	130.0 (2)
C2—C1—H1	119.6	O3—C16—C17	112.7 (2)
C3—C2—C1	120.9 (4)	C15—C16—C17	117.3 (2)
C3—C2—H2	119.6	F3—C17—F2	106.4 (3)
C1—C2—H2	119.6	F3—C17—F1	105.4 (3)
C4—C3—C2	119.1 (3)	F2—C17—F1	105.2 (3)
C4—C3—H3	120.5	F3—C17—C16	112.8 (3)
C2—C3—H3	120.5	F2—C17—C16	111.2 (2)
C3—C4—C5	120.7 (3)	F1—C17—C16	115.2 (2)
C3—C4—H4	119.6	N1—C18—C19	123.2 (3)
C5—C4—H4	119.6	N1—C18—H18	118.4
C6—C5—C4	120.3 (3)	C19—C18—H18	118.4
C6—C5—H5	119.9	C20—C19—C18	119.4 (3)
C4—C5—H5	119.9	C20—C19—H19	120.3
C5—C6—C1	118.2 (3)	C18—C19—H19	120.3
C5—C6—C7	120.3 (3)	C21—C20—C19	118.1 (3)
C1—C6—C7	121.3 (3)	C21—C20—H20	120.9
O1—C7—C6	109.3 (2)	C19—C20—H20	120.9
O1—C7—H7A	109.8	C20—C21—C22	119.6 (3)
C6—C7—H7A	109.8	C20—C21—H21	120.2
O1—C7—H7B	109.8	C22—C21—H21	120.2
C6—C7—H7B	109.8	N1—C22—C21	123.4 (3)
H7A—C7—H7B	108.3	N1—C22—H22	118.3
O1—C8—C9	116.3 (2)	C21—C22—H22	118.3
O1—C8—C13	124.3 (2)	C22—N1—C18	116.3 (2)
C9—C8—C13	119.4 (2)	C22—N1—Co1	119.75 (18)
C10—C9—C8	120.2 (2)	C18—N1—Co1	123.97 (18)
C10—C9—H9	119.9	C8—O1—C7	117.3 (2)
C8—C9—H9	119.9	C14—O2—Co1	127.68 (15)
C9—C10—C11	121.1 (2)	C16—O3—Co1	121.67 (15)
C9—C10—H10	119.4		
			
C6—C1—C2—C3	0.7 (6)	C18—C19—C20—C21	0.7 (5)
C1—C2—C3—C4	−0.3 (6)	C19—C20—C21—C22	−0.8 (5)
C2—C3—C4—C5	0.1 (6)	C20—C21—C22—N1	0.7 (5)
C3—C4—C5—C6	−0.4 (6)	C21—C22—N1—C18	−0.3 (4)
C4—C5—C6—C1	0.9 (5)	C21—C22—N1—Co1	179.1 (2)
C4—C5—C6—C7	177.3 (3)	C19—C18—N1—C22	0.2 (4)
C2—C1—C6—C5	−1.0 (5)	C19—C18—N1—Co1	−179.3 (2)
C2—C1—C6—C7	−177.4 (3)	O2—Co1—N1—C22	−169.9 (2)
C5—C6—C7—O1	122.3 (3)	O2^i^—Co1—N1—C22	12.5 (2)
C1—C6—C7—O1	−61.4 (4)	O3—Co1—N1—C22	176.8 (11)
O1—C8—C9—C10	−175.8 (2)	O3^i^—Co1—N1—C22	99.3 (2)
C13—C8—C9—C10	2.2 (4)	N1^i^—Co1—N1—C22	−77.3 (2)
C8—C9—C10—C11	0.5 (4)	O2—Co1—N1—C18	9.5 (2)
C9—C10—C11—C12	−2.4 (4)	O2^i^—Co1—N1—C18	−168.1 (2)
C9—C10—C11—C14	173.7 (2)	O3—Co1—N1—C18	−3.8 (13)
C10—C11—C12—C13	1.7 (4)	O3^i^—Co1—N1—C18	−81.3 (2)
C14—C11—C12—C13	−174.6 (2)	N1^i^—Co1—N1—C18	102.1 (2)
C11—C12—C13—C8	1.0 (4)	C9—C8—O1—C7	179.2 (2)
O1—C8—C13—C12	174.9 (3)	C13—C8—O1—C7	1.3 (4)
C9—C8—C13—C12	−2.9 (4)	C6—C7—O1—C8	−177.0 (2)
C12—C11—C14—O2	20.2 (3)	C15—C14—O2—Co1	−17.7 (3)
C10—C11—C14—O2	−155.8 (2)	C11—C14—O2—Co1	163.27 (16)
C12—C11—C14—C15	−158.8 (2)	O2^i^—Co1—O2—C14	−17.4 (2)
C10—C11—C14—C15	25.2 (4)	O3—Co1—O2—C14	25.9 (2)
O2—C14—C15—C16	−1.3 (4)	O3^i^—Co1—O2—C14	−60.6 (2)
C11—C14—C15—C16	177.7 (2)	N1^i^—Co1—O2—C14	118.5 (2)
C14—C15—C16—O3	0.0 (5)	N1—Co1—O2—C14	−153.3 (2)
C14—C15—C16—C17	−179.4 (2)	C15—C16—O3—Co1	18.4 (4)
O3—C16—C17—F3	−50.1 (3)	C17—C16—O3—Co1	−162.15 (18)
C15—C16—C17—F3	129.5 (3)	O2—Co1—O3—C16	−24.7 (2)
O3—C16—C17—F2	69.4 (3)	O2^i^—Co1—O3—C16	152.9 (2)
C15—C16—C17—F2	−111.1 (3)	O3^i^—Co1—O3—C16	66.22 (18)
O3—C16—C17—F1	−171.1 (2)	N1^i^—Co1—O3—C16	−117.23 (19)
C15—C16—C17—F1	8.5 (4)	N1—Co1—O3—C16	−11.5 (13)
N1—C18—C19—C20	−0.4 (5)		

Symmetry codes: (i) −*x*+1, *y*, −*z*+3/2.

### Hydrogen-bond geometry (Å, °)

**Table:**

*D*—H···*A*	*D*—H	H···*A*	*D*···*A*	*D*—H···*A*
C2—H2···F2^ii^	0.93	2.62	3.385 (3)	140.
C19—H19···F3^iii^	0.93	2.63	3.348 (3)	134.
C7—H7B···F1^iii^	0.97	2.62	3.507 (3)	152.

Symmetry codes: (ii) *x*−1/2, −*y*+5/2, *z*+1/2; (iii) *x*−1/2, *y*−1/2, *z*.

## References

[bb1] Bruker (1999). *SAINT* Bruker AXS Inc., Madison, Wisconsin, USA.

[bb2] Bruker (2001). *SMART* Bruker AXS Inc., Madison, Wisconsin, USA.

[bb3] Feng, Y.-L. (2002). *Chin. J. Inorg. Chem.* **18**, 723–725.

[bb4] Melnik, M., Kabesova, M., Koman, M., Macaskova, L. & Holloway, C. E. (1999). *J. Coord. Chem.* **48**, 271–374.

[bb5] Sheldrick, G. M. (1996). *SADABS* University of Göttingen, Germany.

[bb6] Sheldrick, G. M. (2008). *Acta Cryst.* A**64**, 112–122. 10.1107/S0108767307043930 18156677

[bb7] Soldatov, D. V., Tinnemans, P., Enright, G. D., Ratcliff, C. I., Diamente, P. R. & Ripmeester, J. A. (2003). *Chem. Mater.* **15**, 3826–3840.

[bb8] Youngme, S., Chotkhun, T., Chaichit, N., van Albada, G. A. & Reedijk, J. (2007). *Inorg. Chem. Commun.* **10**, 843–848.

